# Cryptic *FMR1* mosaic deletion in a phenotypically normal mother of a boy with fragile X syndrome: case report

**DOI:** 10.1186/s12881-014-0125-2

**Published:** 2014-11-25

**Authors:** Shiyu Luo, Wen Huang, Qiuping Xia, Yan Xia, Qian Du, Lingqian Wu, Ranhui Duan

**Affiliations:** The State Key Laboratory of Medical Genetics & School of Life Sciences, Central South University, Changsha, 410078 Hunan China

**Keywords:** Deletion, Fragile X syndrome, Mosaic, Somatic heterogeneity

## Abstract

**Background:**

Increasing number of case reports of mosaic mutations and deletions have better armed clinicians and geneticists with more accurate and focused prenatal diagnoses. Since mosaicism means a significant increase of recurrence risk, detailed parental profiling is essential for risk assessments.

**Case presentation:**

We here describe a clinically unaffected mother with a son who had fragile X syndrome (FXS) caused by a large deletion that includes the entire *FMR1*. To assess the recurrence risk regarding her second pregnancy, a series of genetic tests were conducted to establish this mother’s status. Routine single nucleotide polymorphism (SNP) array and fluorescence in situ hybridisation (FISH) analyses detected two normal *FMR1* copies in her blood. However, in-depth studies across the deleted region revealed varying proportions of mosaic deletion in her somatic tissues: lowest in the blood, moderately higher in the skin, urine sediment and menstrual discharge and highest in her eyebrow. Further FISH analysis of her skin-derived fibroblasts confirmed mosaicism of 13%.

**Conclusion:**

To our knowledge, this is the first characterized case of a female who was mosaic for an *FMR1* deletion and extensive investigation of her mosaic status provided valuable information for her reproduction choices. Our case report may also alert clinicians and geneticists that a cryptic mosaicism with somatic heterogeneity should be carefully considered in families with children having clinically defined ‘de novo’ mutations, to avoid a second pregnancy with identical genetic abnormalities.

## Background

Fragile X syndrome (FXS) is the most common cause of inherited intellectual disability, with an estimated prevalence of 1 in 4 000 males. Over 95% of the cases of FXS are caused by an expansion of a CGG trinucleotide repeat in the 5′ untranslated region of the fragile X mental retardation gene 1(*FMR1*) [1]. Point mutations and deletions of several bases to megabases have also been reported to result in mental retardation, developmental delays and other fragile X features in the remaining 5% of cases [2,3]. Among 22 deletions involving part or the entire *FMR1*, the majority (15 cases) reportedly occurred de novo, 4 were maternally inherited, 1 had a mosaic *FMR1* deletion and 2 had mosaicisms for a deletion and repeat expansion probably caused by post-zygotic CGG instability.

The increasing number of case reports of mosaic mutations and deletions has made clinicians and geneticists more aware of the necessity of more accurate prenatal diagnoses. Mosaicism with large genomic deletions has been consistently identified as an underlying mechanism in patients who tested negative by routine mutational screening for causal genes [4]. Mosaic deletions may arise de novo or be passed on by phenotypically normal mosaic parents [5]. For example, evidence of parental germline mosaicism was provided in a family with two siblings who carried an identical deletion at 19p13.13, which was absent in both of the parents [6]. Apparently, parental mosaicism at a low level and/or tissue heterogeneity may be easily misdiagnosed as de novo transmission with routine genetic tests. Thus, detailed, in-depth parental profiling is necessary for accurate risk assessment in families with clinically defined de novo mutations.

We present a case of a phenotypically normal mother with a son who had FXS, in whom a mosaic *FMR1* deletion with somatic heterogeneity was identified by sensitive qPCR analysis targeting the breakpoints and comprehensive examination of multiple tissue samples. Because her first born son had FXS and this mother had a heterogeneous mosaic deletion, prenatal diagnosis is recommended to exclude recurrence. A test that exclusively targets these deletion breakpoints was also established to accurately distinguish the normal genotype from either a deleted hemizygote or a heterozygote.

## Case presentation

A typical FXS boy was previously identified to be carrying a large deletion that encompassed the entire *FMR1* by screening a cohort of Chinese paediatric patients with suspected FXS [7]. No family history of mental retardation was noted. His phenotypically normal, 30-year-old mother was concerned about her future pregnancies, and thus requested genetic counselling regarding her second pregnancy.

## Methods

### Molecular analysis

Genomic DNA was extracted from peripheral blood using a standard phenol-chloroform method and then used for *FMR1* exon amplification and SNP array analysis using Illumina HumanCytoSNP-12 v2.1 BeadChip (Illumina, San Diego, California, US), according to the manufacturer’s instructions. The data from the images acquired were analysed using cnvPartition Plugin v3.1.6 for GenomeStudio.

### FISH analysis

FISH analysis was separately performed for leukocytes and skin-derived fibroblasts. In routine analysis, 100 counts of mitoses and nuclei were scored for normal or abnormal FISH signals. However, based on the very low level of mosaicism detected in the blood of this mother, 1000 counts were used. The deleted region was investigated using a BAC clone Rp11-161 L9 mapped to chromosome Xq27.3-Xq28 (chrX: 146 996 078–147 161 084) and labelled with Spectrum orange-dUTP, and with Rp11-54I20 mapped to Xq28 (chrX: 152 706 073–152 898 056) and Rp11-93 M8 mapped to X p11.21 (chrX: 57 177 462–57 293 822) labelled with Spectrum green-dUTP as controls.

### *FMR1* dosage evaluation

To evaluate the *FMR1* dosage in this mother, quantitative real time PCR was conducted using a C1000 Touch thermal cycler (Bio-Rad, Hercules, CA, USA). Primer pairs were designed to be within the deletion region (5′-F: 5′-GAATGAGAGGTCATGGTTAAAGGA-3′, 5′-R: 5′-ACCCAGCTGAAATGCCTTCT-3′; Exon7-F: 5′-GGCAGCTTGCCTCGAGATTT-3′, Exon7-R: 5′-GCAGTGACCCCAGGTACTTT-3′; Exon15-F: 5′-GCAGTTGCGACAGATTGGAG-3′, Exon15-R: 5′-ACCTCGACCCATTCCTTGAC-3′). *COBL* was chosen as a reference gene. Target and reference gene amplifications were done with a total volume of 20 μL that included 0.2 μM of each primer, 20 ng of genomic DNA and 1x FastStart Essential DNA Green Master (Roche, Penzberg, Upper Bavaria, Germany). The amplification conditions were: 10 min pre-incubation at 95°C, followed by 40 cycles of 10 sec at 95°C and 30 sec at 60°C. To confirm the specificity of an amplified product, a default melting program was run at the end of the cycling program. All samples were tested in triplicate three times each. The dosage of the targeted fragments was calculated using the 2 − ΔΔCT method and analysed using GraphPad Prism 5 software (GraphPad Software, Inc., La Jolla, CA, USA). The dosage of a normal male was set at 1.

### Deletion breakpoints mapping

To determine the exact deletion breakpoints, *FMR1* neighbouring genomic sequences in Xq27.3-Xq28 were sequentially mapped by PCR-electrophoreses for the hemizygous son. Primers and conditions used are available upon request. Deletion breakpoints were ultimately identified by sequencing a PCR product of about 550 bp across the deleted region, with primers F1 (5′-AGTTTACAGGAGCCTTATTCAT-3′) and R1 (5′-CTTCCCACCAACTAGACAAT-3′) flanking these breakpoints.

### Dosage evaluation of deleted alleles in multiple tissues from the mother

Tissue samples were collected from the mother, including a buccal swab, urine sediment (containing bladder epithelial cells and some amount of menstrual blood), menstrual discharge (mixture of menstrual blood and mucosal tissue from the inner lining of the uterus), eyebrow (10 drops with a visible hair bulb) and skin (biopsy performed by physician specialist). A small part of the skin tissue was used directly for DNA extraction and the remainder was used for fibroblast culture. DNA was isolated from the menstrual discharge and skin tissue using a standard phenol-chloroform method. DNA extraction from the eyebrow, buccal swab and urine sediment was done using a QIAamp DNA Mini Kit (Qiagen, Hilden, Germany), according to the instructions provided in the QIAamp DNA Mini and Blood Mini Handbook.

Quantifying the deleted alleles in the mother’s multiple tissues was done by qPCR using a C1000 Touch thermal cycler (Bio-Rad, Hercules, CA, USA). The primer pair that flanked the deletion breakpoints was: F2: 5′-ACTGAAAGCAACCAAGA-3′, R2: 5′-TGTGAAAGAAACTGCTGAG-3′, with *COBL* as a reference gene. qPCR was conducted with a total volume of 20 μL that included 0.2 μM of each primer, 20 ng of genomic DNA and 1× FastStart Essential DNA Green Master (Roche, Penzberg, Upper Bavaria, Germany). The amplification conditions were: 10 min pre-incubation at 95°C, followed by 40 cycles of 10 sec at 95°C and 30 sec at 60°C. To confirm the specificity of an amplified product, a default melting program was run at the end of the cycling program. All samples were tested in triplicate and repeated three times each. The dosages of the deleted alleles among different tissue samples from the mother were calculated using the 2 − ΔΔCT method and analysed using GraphPad Prism 5 software (GraphPad Software, Inc., La Jolla, CA, USA). The dosage of the hemizygous proband was set at 100%.

### Molecular study results

**Figure 1:**
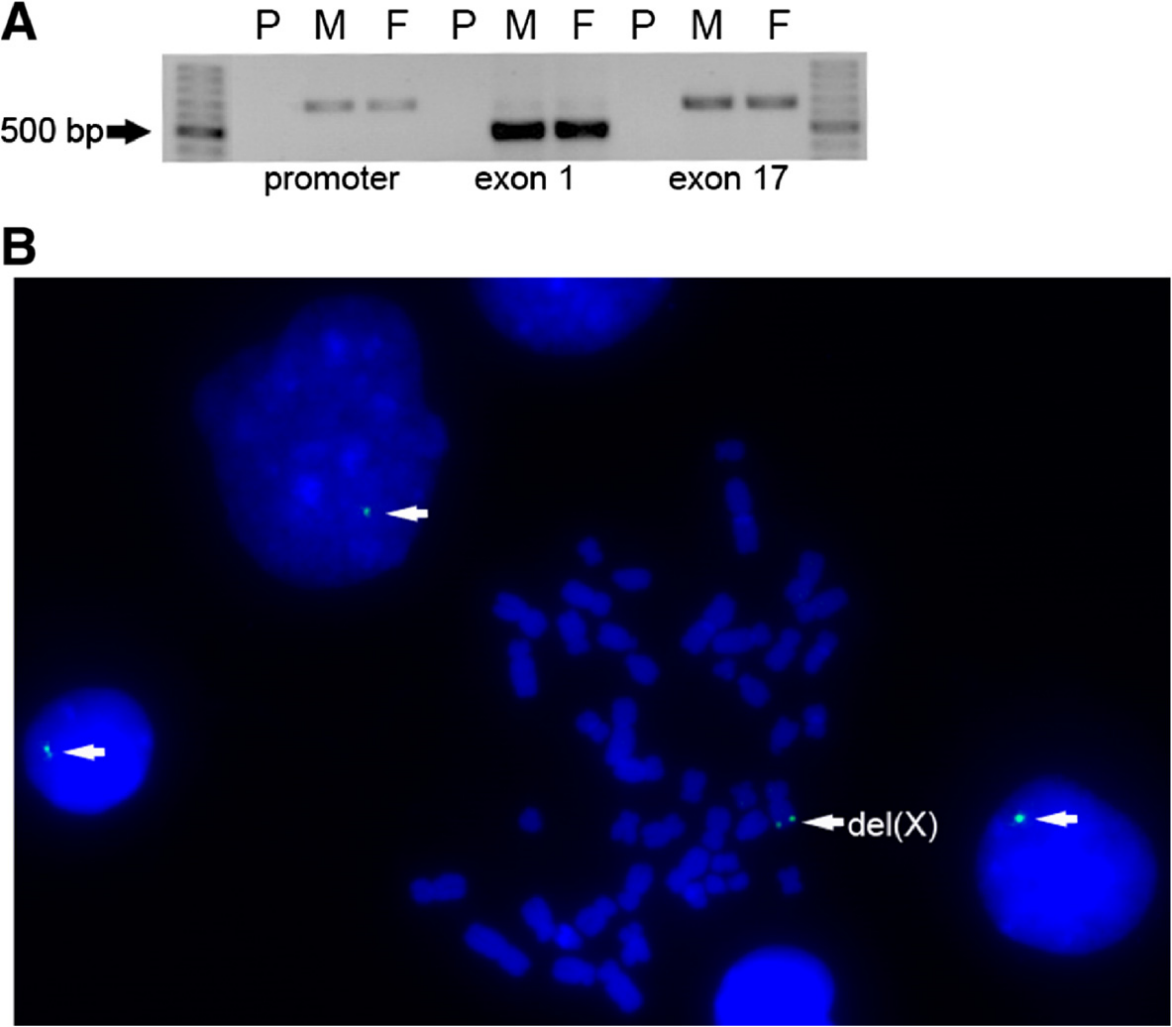
**Large deletion identified in a typical boy with FXS. (A)** Representative electrophoresis results for the PCR products of *FMR1* for the proband and his normal parents. No products were obtained for the proband, which indicated a large deletion that covered the entire *FMR1*. P: proband, M: mother, F: father. **(B)** FISH analysis for leukocytes from the proband using Rp11-161 L9 and Rp11-54I20. As indicated by the white arrows, only the green control signals were observed in the mitoses and nuclei, whereas the targeted orange signal was missing. del(X): X chromosome carrying the deletion.

Screening for *FMR1* mutations in a cohort of paediatric patients with suspected FXS identified a large deletion in a boy with moderate intellectual disability and marked hyperactivity. Routine PCR amplification of the CGG repeats and Southern blot analysis using a StB12.3 probe detected no signals (data not shown), which indicated no sequences that were complementary to this probe. Attempting to amplify all regions in *FMR1* failed, which established a deletion for the entire gene (Figure 1A). Consist with these results, FISH analysis using leukocytes from the proband only showed a green control signal without the targeted orange signal (Figure 1B).

**Figure 2:**
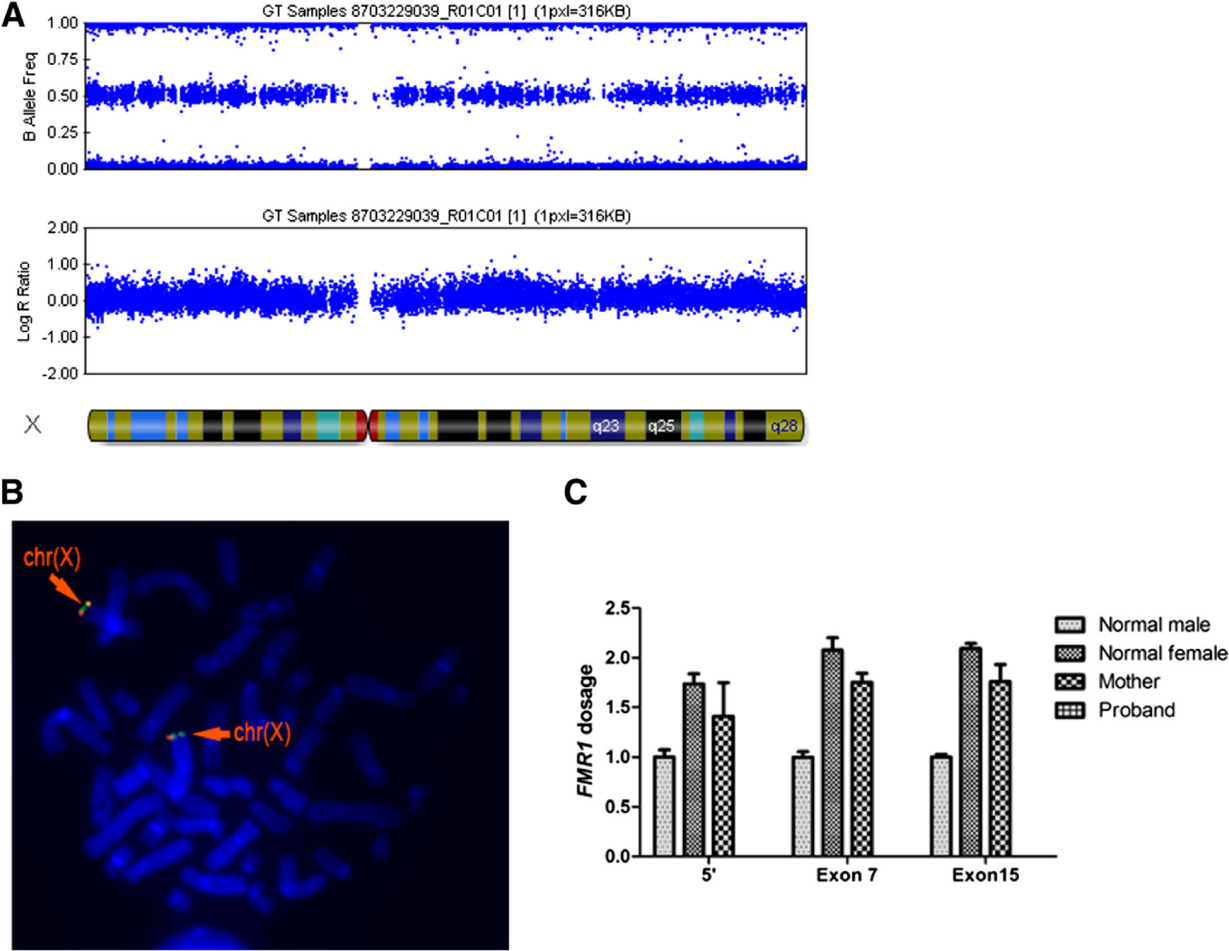
**Genetic tests for the mother showed two normal** ***FMR1*** **copies in her blood. (A)** Genome-wide copy number analysis for the mother, showing no deleted region on the X chromosomes in her blood based on the B allele frequency and log R ratio. **(B)** FISH using Rp11-161 L9 and Rp11-54I20 detected both the targeted orange signal and the green control signal on the mother’s two X chromosomes, as indicated by the orange arrows. chr(X): normal X chromosome. **(C)** Sensitive qPCR analysis for *FMR1* dosage using three sets of primers within the deleted region showed no significant difference between the mother and a normal female control. Error bars indicate standard deviations. 5′: with primers 5′-F and 5′-R, Exon 7: with primers Exon7-F and Exon7-R, Exon 15: with primers Exon 15-F and Exon 15-R.

The mother, who was phenotypically normal, was concerned regarding her future pregnancy; thus, she had requested genetic counselling for a risk assessment. Genome-wide copy number analysis for the mother found no deleted region on the X chromosomes in her blood (Figure 2A). FISH analysis using her leukocytes detected both the targeted orange signal and the green control signal on the mother’s two X chromosomes (Figure 2B). To assess the *FMR1* dosage for the mother, sensitive qPCR analyses were run using three primer sets located within the deleted region. As expected, all PCR reactions failed with a sample from the proband due to the loss of the entire *FMR1*. qPCR analyses were repeated three times and consistently showed 15%-20% decreases in the amplification products of the mother as compared to a female control using all of the targeted primer sets (5′, Exon 7 and Exon 15 normalised by *COBL*). This decreased proportion did not reach the defined threshold for qPCR analysis (30%) and was not considered as a significant variation (Figure 2C).

**Figure 3:**
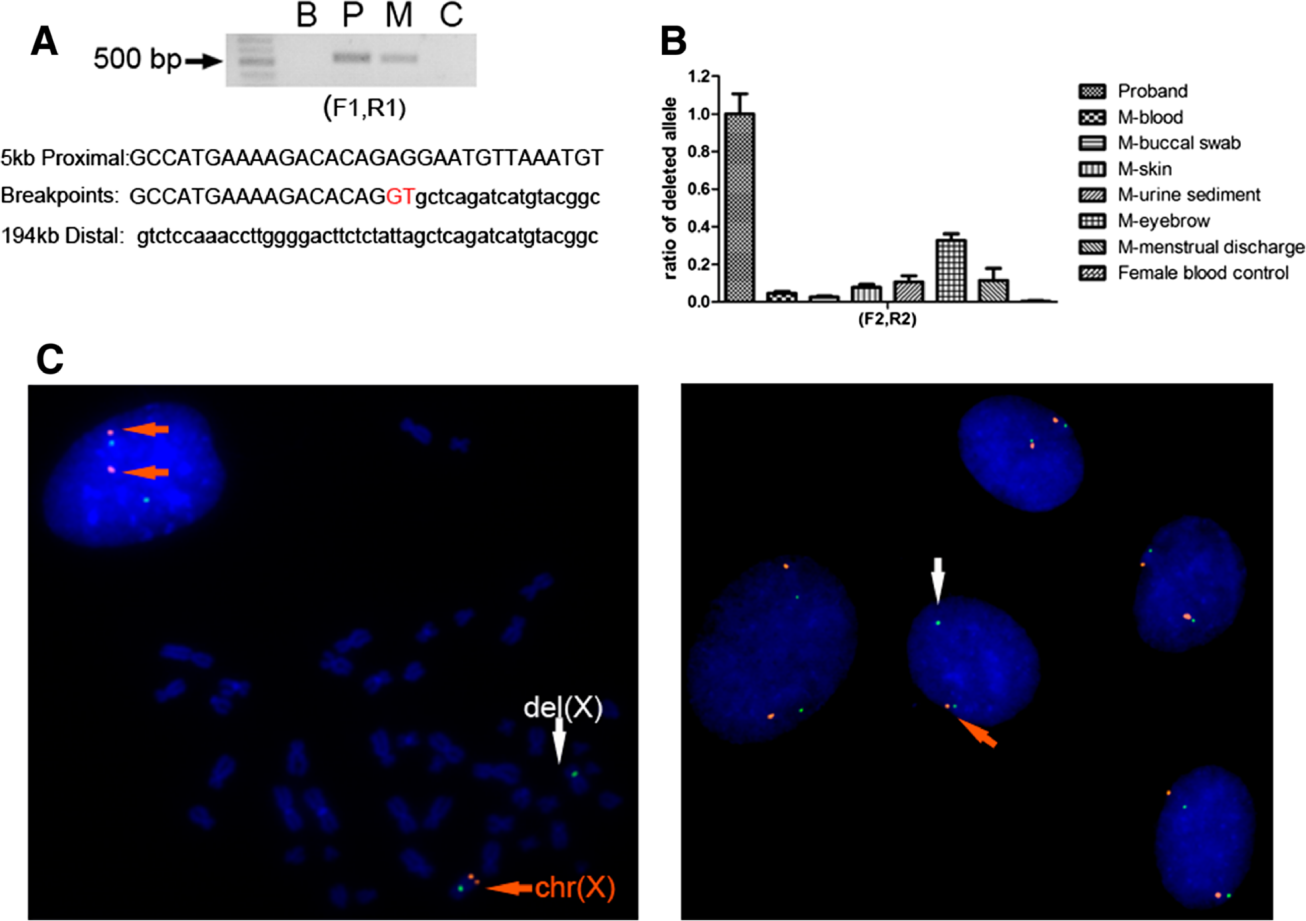
**Varying proportions of a mosaic deletion among different tissue samples from the mother. (A)** Breakpoint analysis for the deletion in the proband and his mother. Upper panel: Electrophoresis results for the amplified products with primers F1 and R1, which show bands of about 550 bp for both the proband and his mother. This amplification failed for normal controls due to the huge span, suggesting a same deletion in the mother and in her son. B: blank control, P: proband, M: mother, C: normal female control. Bottom panel: Sequence analyses of the breakpoints in the proband and the mother revealed an insertion (red) of two nucleotides (GT) at the junction. This deletion extended approximately 5 kb proximal to and 194 kb distal of the *FMR1*. **(B)** qPCR analyses of the deleted alleles show varying proportions of mosaicism among multiple tissues from the mother, including her eyebrow, buccal swab, skin, urine sediment and menstrual discharge. The mosaic proportion of the deleted alleles was low in the blood (4%), while it was higher in the skin (8%), urine sediment (11%), menstrual discharge (12%) and eyebrow (33%). Error bars indicate standard deviations. **(C)** Representative FISH analysis of the mother’s skin-derived fibroblasts. Left panel shows an abnormal mitosis with one X chromosome that has only the green control signal (indicated by the white arrow), suggesting a loss of the complementary fragment in this cell. Neighbouring nuclei had both targeted and control signals on both X chromosomes, suggesting a normal genotype. Right panel shows an abnormal nucleus (indicated by the white arrow) surrounded by four nuclei with normal genotype. A total of 213 abnormal cells (9 mitoses and 204 nuclei) with single targeted signal and 2 control signals were detected based in 1600 counts, which indicated mosaicism of 13% in the mother’s fibroblasts.

To entirely exclude the possibility of a mosaic deletion in the mother, the exact breakpoint was mapped and PCR amplification was done using primers (F1 and R1) that flanked the deletion breakpoints. Interestingly, amplified products of similar length (about 550 bp) were obtained using blood samples from this mother and her son, which failed with normal control DNA due to the huge span (Figure 3A, upper panel). Sequencing of these amplified products further revealed that the same breakpoints were present in both mother and son. As shown in the bottom panel of Figure 3A, this deletion was found to extend approximately 5 kb proximal to and 194 kb distal of *FMR1*, with an insertion of two nucleotides (‘GT’) between the deletion junctions. It was noteworthy that these breakpoints were located within two non-LTR retrotransposons, a long interspersed element (*L1MC2*) and a short interspersed element (*MIR3*), respectively, although no sequence homology was observed between the proximal and distal breakpoints.

To verify the presence of this deletion in the mother, 1000 FISH counts using her leukocytes were made. Ultimately, 4 abnormal nuclei with 2 control signals and a single targeted signal were detected (data not shown). Given the technical limitations with FISH, the 4 in 1000 abnormal cells observed in the mother’s leukocytes, which was far below the clinical standard for defining mosaicism (3%), may have been background signals. However, we could not exclude the possibility of a low level mosaicism for the deletion, which could have resulted in successful amplification across the deleted region in the mother’s blood.

Because of the inconsistency between the amplifications across the breakpoints and FISH results using this mother’s blood, we collected tissue samples, including a buccal swab, urine sediment, menstrual discharge, eyebrow and skin to explore her status in somatic tissues. qPCR analyses were done using primers (F2 and R2) that flanked these breakpoints. As shown in Figure 3B, the mosaic proportion of deleted alleles, which was defined as 100% for the hemizygous son, was quite low in her blood (4%), whereas it was higher in her skin (8%), urine sediment (11%), menstrual discharge (12%) and eyebrow (33%). The most significant variation was up to 8-fold higher when comparing her blood and eyebrow samples.

To authenticate the mosaic status of this mother, FISH analysis was done using her skin-derived fibroblasts. A total of 213 abnormal cells (9 mitoses and 204 nuclei) with 2 control signals and a single targeted signal were detected based on 1600 counts (Figure 3C). The proportion of fibroblasts that carried the mosaic deletion was close to 13%, which indicated that the proportion of deleted alleles was 6.5%. Thus, we concluded that this mother had a deletion mosaicism with tissue heterogeneity.

## Discussion

Techniques that include common chromosome analysis, array-comparative genomic hybridization (CGH), genome-wide SNP array, FISH and multiple Southern blots have been used to identify gross gene deletions [2,3,8-10]. A major disadvantage of these techniques is their limited ability to detect a mosaicism, which may be occasionally encountered in clinical practice. No specific molecular strategy has been devised to detect a low level of diverse genetic alterations, including point mutations, intragenic or large genomic deletions, duplications and translocations. Here we identified a female with a normal phenotype who carried a mosaic *FMR1* deletion with varying proportions in her blood and other tissues. Since the presence of mosaicism results in an increased transmission risk, the localization of deletion breakpoints in affected individuals is required for parental evaluations and accurate individualized prenatal diagnosis using qPCR analysis across the deleted region.

For the first time, we identified a female with a mosaicism for an *FMR1* deletion with somatic heterogeneity. The mosaic proportion of the deleted alleles in this mother was lowest in her blood (mesoderm-derived), moderately higher in her skin and urine sediment (mixture of ectoderm- and mesoderm-derived) and menstrual discharge that contained blood and uterine mucosal tissue (mixture of mostly mesoderm- and endoderm-derived) and was highest in her eyebrow (ectoderm-derived). It has been demonstrated that in human embryos, primordial germ cells (PGC) can be found in the epiblast (primary ectoderm) at the second week and then migrate from the primary ectoderm into the yolk sac wall during the third week. At around the 16^th^ day after fertilisation, gastrulation occurs to re-organize the two-layer embryo into a three-layer embryo, with the epiblast differentiating into the three germ layers of the embryo and the hypoblast to form the amnion [11]. In our case, the deletion should have occurred early in the epiblast before germ layer differentiation and PGC migration, considering the ubiquitous distribution of two different cell lines in multiple tissues from this mother and the germ-line transmission of the deletion to her son. The mosaic proportion in the ectoderm tends to be higher than that in the mesoderm, which resulted in the higher mosaic proportion in her eyebrow than in her blood. Since the inaccessibility of female germ cells makes an accurate risk assessment impossible, extensive examination of multiple tissue samples is highly recommended.

## Conclusions

Comprehensive evaluation of tissue samples derived from different germ layers revealed a mosaic deletion of varying proportions in this mother. The low-level of deleted alleles in blood would have been undetectable during routine clinical tests, which may have resulted in a misdiagnosis of the deletion in her son as being de novo. Based on these circumstances, obligatory prenatal tests are required to verify the normality of the fetus. Pre-implantation genetic diagnosis (PGD) is also an alternative and may provide the actual germ line status. It should be emphasized that mosaicism at a low level and somatic heterogeneity should be considered with great caution for parents who have had affected children.

## Consent

This study was approved by the Ethics Committee of the State Key Laboratory of Medical Genetics, Central South University (Approval ID: 2013051203). Written informed consent was obtained from the mother of the patient for publication of this case report and any accompanying images. A copy of the written consent is available for review by the Editor of this journal.

## Abbreviations

CGH: Comparative genomic hybridization
FISH: Fluorescence in situ hybridization
*FMR1*: Fragile X mental retardation gene 1
FXS: Fragile X syndrome
PGC: Primordial germ cells
PGD: Pre-implantation genetic diagnosis
SNP: Single nucleotide polymorphism
